# New York

**Published:** 1896-10

**Authors:** 


*New York.* The Health Department of New York City, between July 31st and September 14th, inspected 153 cows, 28 of which were found to be tuberculous and were destroyed. One hundred and seventy-eight families, or about five hundred persons, received milk from these animals. It is expected that the work will require a period of five or six weeks to cover the entire State, in which there is estimated to be 2500 cattle; the number already tested is 2147, of which 405, or nearly 20 per cent., were condemned. The quality of the food- and water-supply to the cows of New York City is also being examined into at the same time.

Tuberculosis has caused 61,155 deaths in the human family in the last ten years in the city of New York.

New York City claims the power to go out into the State, if necessary, to insure a pure milk-supply to the city, and purposes exercising this power.

Twenty thousand of New York's population, it is estimated, receive milk from cows kept within the city limits.

